# Newly discovered clouting interplay between matrix metalloproteinases structures and novel quaternary Ammonium K21: computational and in-vivo testing

**DOI:** 10.1186/s12903-024-04069-0

**Published:** 2024-03-25

**Authors:** Ranjeet Ajit Bapat, Kit-Kay Mak, Mallikarjuna Rao Pichika, Jia Chern Pang, Seow Liang Lin, Suan Phaik Khoo, Umer Daood

**Affiliations:** 1 Restorative Dentistry Division, School of Dentistry, International Medical University Kuala Lumpur, Kuala Lumpur, Malaysia; 2 School of Pharmacy, International Medical University Kuala Lumpur, 126, Jalan Jalil Perkasa 19, Bukit Jalil, 57000 Wilayah Persekutuan Kuala Lumpur, Malaysia; 3 School of Postgraduate Studies, International Medical University, 57000 Kuala Lumpur, Malaysia; 4 Division of Clinical Oral Health, School of Dentistry, International Medical University, Kuala Lumpur, Malaysia

**Keywords:** QAS, K21, S^1^ Binding, Antimicrobial, Cell death, Osmoregulation, Proteases, MMPs

## Abstract

**Aims and objectives:**

To analyze anti-MMP mode of action of Quaternary Ammonium Silane (QAS, codenamed as k21) by binding onto specific MMP site using computational molecular simulation and Anti-Sortase A (SrtA) mode of action by binding onto specific site using computational molecular simulation.

**Materials and methods:**

In silico Molecular Dynamics (MD) was used to determine the interactions of K21 inside the pocket of the targeted protein (crystal structure of fibroblast collagenase-1 complexed to a diphenyl-ether sulphone based hydroxamic acid; PDB ID: 966C; Crystal structure of MMP-2 active site mutant in complex with APP-derived decapeptide inhibitor. MD simulations were accomplished with the Desmond package in Schrödinger Drug Discovery Suite. Blood samples (~ 0.5 mL) collected into K_2_EDTA were immediately transferred for further processing using the Litron MicroFlow® PLUS micronucleus analysis kit for mouse blood according to the manufacturer’s instructions. Bacterial Reverse Mutation Test of K21 Molecule was performed to evaluate K21 and any possible metabolites for their potential to induce point mutations in amino acid-requiring strains of *Escherichia coli (E. coli) (*WP2 *uvrA* (tryptophan-deficient)*).*

**Results:**

Molecular Simulation depicted that K21 has a specific pocket binding on various MMPs and SrtA surfaces producing a classical clouting effect. K21 did not induce micronuclei, which are the result of chromosomal damage or damage to the mitotic apparatus, in the peripheral blood reticulocytes of male and female CD-1 mice when administered by oral gavage up to the maximum recommended dose of 2000 mg/kg. The test item, K21, was not mutagenic to Salmonella *typhimurium (S. typhimurium)* strains TA98, TA100, TA1535 and TA1537 and *E. coli* strain WP2 *uvrA* in the absence and presence of metabolic activation when tested up to the limit of cytotoxicity or solubility under the conditions of the test.

**Conclusion:**

K21 could serve as a potent protease inhibitor maintaining the physical and biochemical properties of dental structures.

**Supplementary Information:**

The online version contains supplementary material available at 10.1186/s12903-024-04069-0.

## Background and summary

Dental diseases are initiated by anaerobic bacteria present in dental plaque, that initiates host response involving complex network of proinflammatory mediators like growth factors, cytokines, and matrix metalloproteinases (MMPs) [1]. During physiologic and pathologic processes, changes in extracellular matrix (ECM) are regulated by various protease enzymes i.e., aspartic proteinase, serine proteinase, cysteine proteinase and metalloproteinase. MMPs cause destruction of connective tissue resulting in Periodontitis. Amongst various superfamilies of metalloproteinases, MMPs belong to metzincin superfamily [2]. There are several groups of MMPs like MMPs 1, 8, 13 (collagenases), MMPs 2 and 9 (gelatinase), MMPs 3, 10 and 11 (stromelysin), MMP7 (matrilysins) and other membrane associated MMPs [2, 3].

**Fig. 1:**
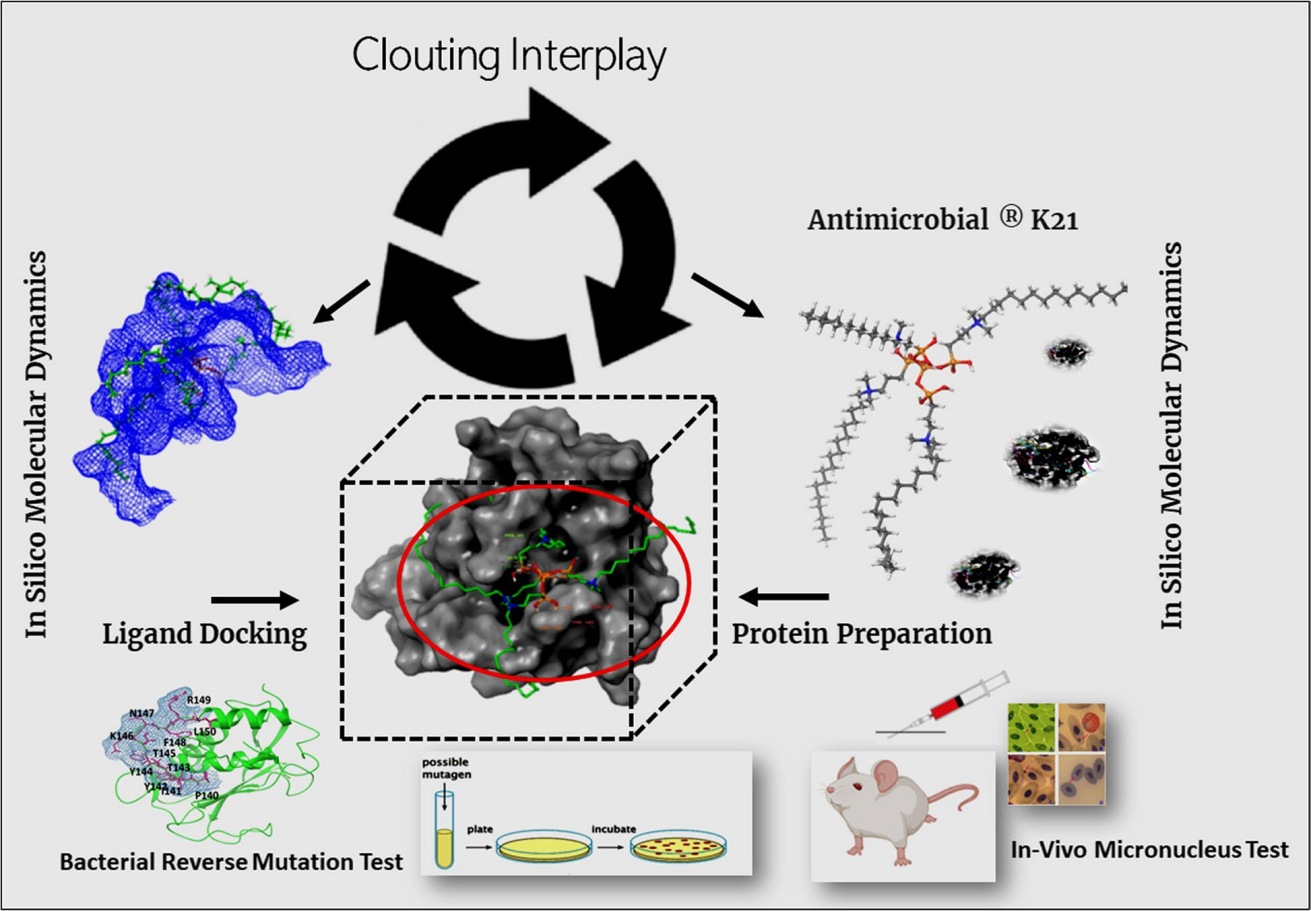
Schematic representation of experimental design

MMP enzymes stimulate innate and/or adaptive immunity and tissue destruction. Hence, identification and inhibition of MMPs is important as part of prevention or intervention in dentistry. Out of these, MMP1, MMP2, MMP8 and MMP9 (Fig. 1) have been associated with dental diseases [4, 5]. Analyzing the MMPs structure, its active sites have two regions: the center part of cavity is the zinc ion and adjacent site is a specific S1’pocket. Catalytic clefts have S1, S2 and S3 nonprime hydrophobic pockets on left side of zinc ion and S1’, S2’ and S3’ primed pockets on right side of zinc ion S1’ pocket, which is a hydrophobic cavity adjoining active site, has been considered a key factor in designing of MMP inhibitors due its variation in depth, size and based on sequence of amnio acid around S1’ pocket [6]. Thus, S1’s pocket and its surrounding Ω-loop with variable amino acid sequence forms the major basis for MMP substrate recognition and development of MMP inhibitors with selective binding action. Developing selectivity for a specific MMP in relation to drug discovery is an intriguing task. Various MMP inhibitors have displayed extreme adverse effects during research trials due to inadequate selectivity. This is due to their design that exploits features common to all MMPs that includes S1’ pocket, active zinc ions and glutamic acid. Most MMP inhibitors are not capable of targeting specific MMPs related to specific pathology. Instead, they hinder several MMPs, some of which possess preventive functions or are not associated with the pathology [7]. There is a lack of research on selectivity of quaternary ammonium salts attachment on MMP to initiate its anti-MMP action. Although S1’pocket could be the possible target [8], other binding sites like S2’ and S3’ pockets also need to be explored. Hence, the rationale of this research was to recognize the potential catalytic binding sites on MMP for K21 that contribute to substrate distinction and analyzing K21 to be MMPs antagonist. Also assessing attachment with sites other than S1’ pockets, the other areas could also play a key role as future targets to prevent the catalytic action of these enzymes [9].

**Fig. 2:**
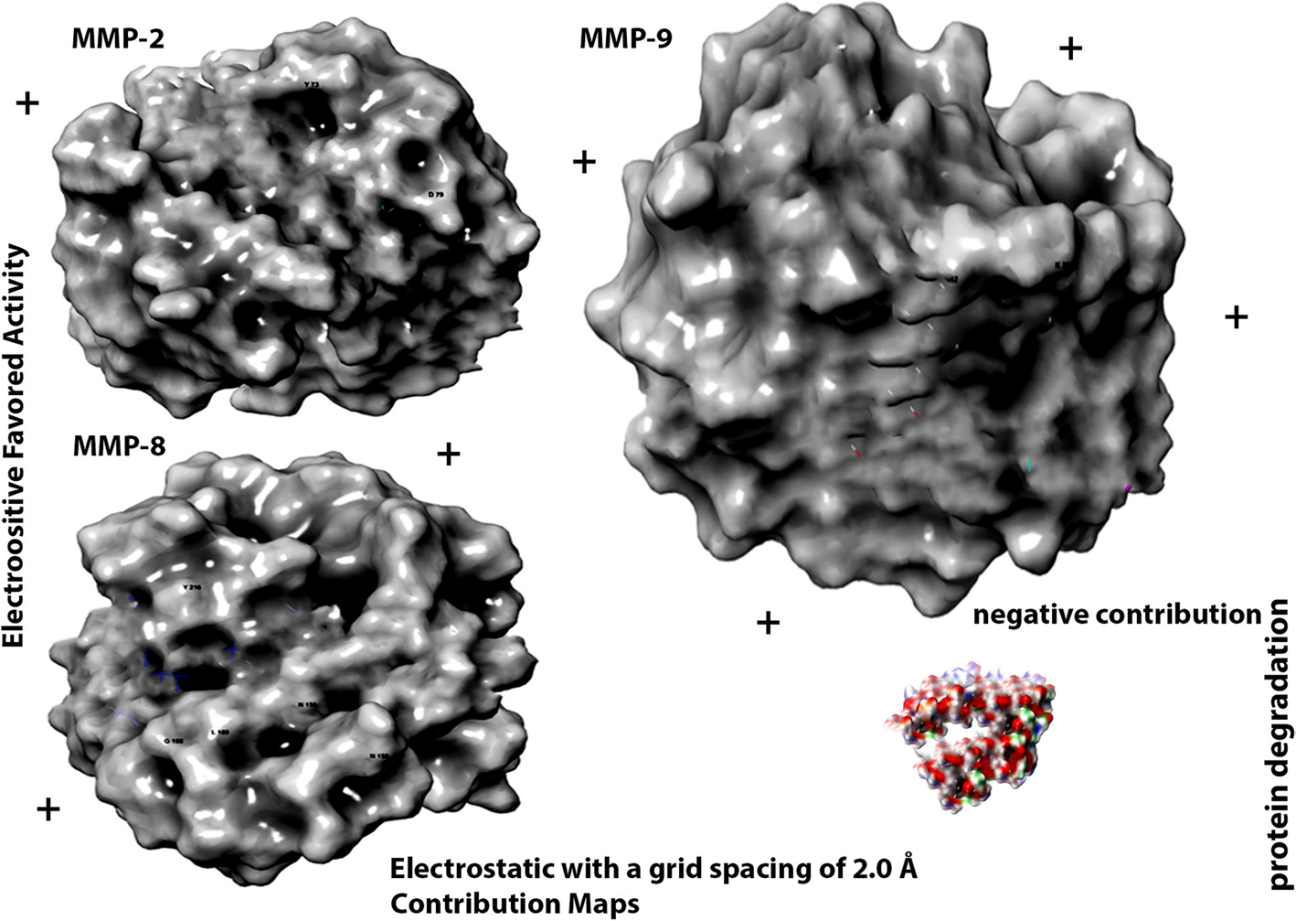
MMP enzymes downloaded through MD simulations rupturing the tissue with hypoxic core which mediates the movement of tissue. RMSD of MD simulations showing degradation conformation for several important proteins via the bonding pose in an explicit water box model simulated for 100 ns

In the caries forming bacteria [10], SrtA enzyme assists in adherence of surface proteins to the cell wall. The SrtA enzyme is coded by SrtA gene, that recognizes LPXTG motif of protein and catalyzes a grouping process to attach protein to cell wall [11]. SrtA plays a critical function in the process by controlling the bacterial adhesion and virulence expression Three-dimensional SrtA also contains an 8-stranded β-barrel structure holding the catalytic pocket (Fig. 2) comprising of the pivotal C184 and H120 residues [12]. This structural conformity is unique and a common feature in all SrtA enzymes which are characterized [13] producing an experimental model for sortase-mediated cell wall anchoring pathway for possible inhibitors. The glaring and interesting part is that the SrtA binding via amino nucleophile, which can originate from a lipid II molecule or the pilin substrate [14]. Thus, agents targeting SrtA enzyme can be beneficial for caries prevention. Applying MD between K21 and SrtA can provide vital inputs on attachment of K21 on SrtA surface at the molecular level (Fig. 2). Thus, the rationale was to elucidate in-depth molecular interaction between K21 and SrtA of hydrophobic and hydrogen interactions that can help in knowing if K21 binds to the active region or other binding zones of SrtA inhibiting biofilm formation.

**Fig. 3:**
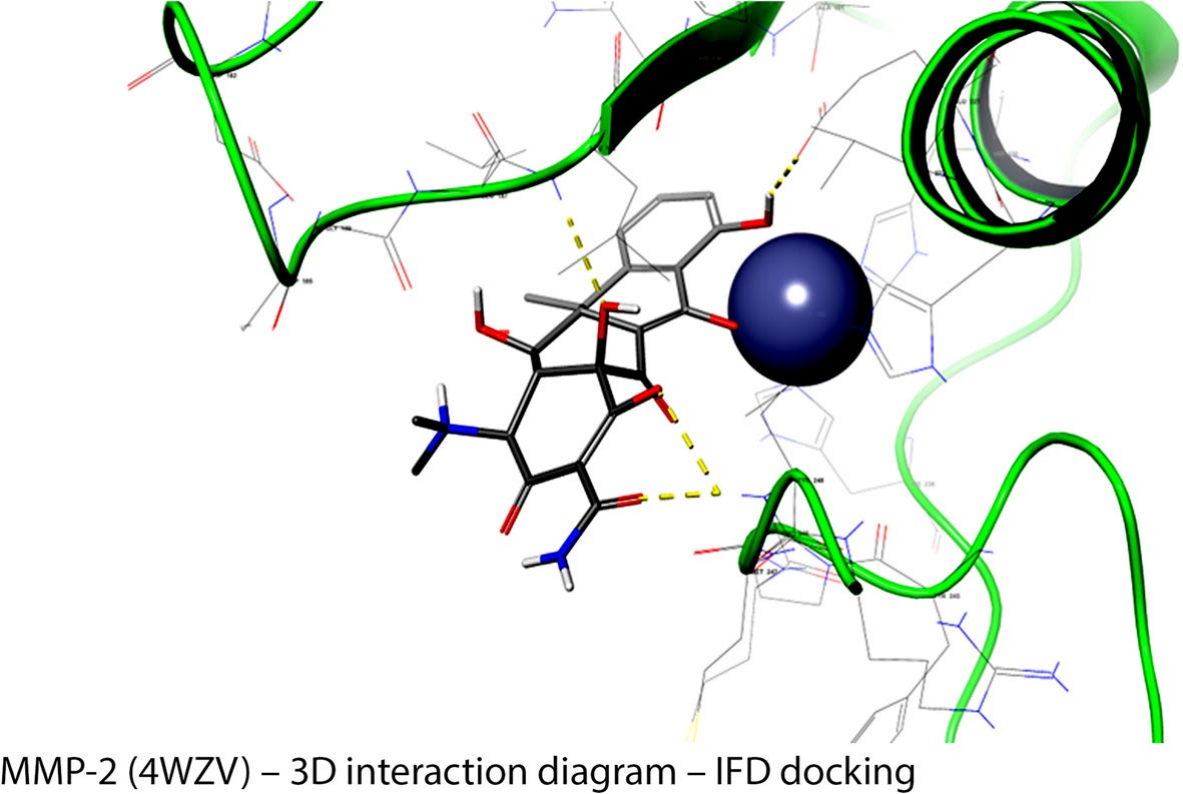
Identifying action of drug of interest by foreseeing ligand-target connections at molecular level and assessing their complementarity by usage of scoring function within MMP-2 (4WZV) – 3D interaction diagram – IFD docking

Quaternary ammonium compounds or K21 (Fig. 3) are surfactants having broad spectrum bactericidal effects [15] penetrating the cell wall of bacteria [16] with interaction with the phospholipid bilayer present within the membranes leaking intracellular components [17]. The compound is based on 1-octadecanaminium, N,N′-[[3,3-bis[[[3-(dimethyloctadecylammonio)propyl] dihydroxysilyl] oxy]-1,1,5,5-tetrahydroxy-1,5-trisiloxanediyl] di-3,1-propanediyl] bis [N,N-dimethyl-], chloride1:4 (C_92_H_2_0_4_Cl_4_N_4_O_12_Si_5_, CAS number 1566577–36-3; code-named K21 (Fig. 2) synthesized via a solgel route using tetraethoxysilane and3-(triethoxysilyl)-propyl dimethyl octadecyl ammonium chloride [18]. K21 prevents secondary caries inhibits resin breaking of resin dentin bonding [19]. K21 displays anti-MMP action by binding onto specific active sites on MMPs [20]. Due to variable expression of S1’ pockets amid all the MMPs, it is assumed that this pocket can be a probable target for MMP inhibitors [21]. The hydrophilic head of K21 absorbs the cell membrane and hydrophobic tails implants itself into lipid layer causing leakage of cytoplasmic contents out of the bacterial cell wall [16]. This leads to structural disorganization of the cell and inhibition of cellular osmoregulation [16]. Thus, K21 has contact killing action by targeting the cell wall, it can be used as anti-adhesion agent by acting as SrtA inhibitor preventing dentin destruction.

Molecular docking is an *in-silico* method to elucidate the binding and interactions between two molecular structures. It allows to identify action of drug of interest by foreseeing ligand-target connections (Fig. 1) at molecular level and assessing their complementarity by usage of scoring function [22]. The aim of the study was to analyze 1] Anti-MMP mode of action of k21 by binding onto specific MMP site using computational molecular simulation and 2] Anti-SrtA mode of action by binding onto specific site using computational molecular simulation. The null hypothesis was 1] K21 binds onto S1’ pocket of MMPs responsible for MMP inhibitory action 2] K21 binds onto SrtA preventing its action using computational molecular simulation.

## Methods

The ethical approval for the project (Fig. 1) was granted by the Institutional Review Board International Medical University (associated with the grants number: IMU R259/2020).

### In silico molecular dynamics

In silico MD was used to determine the interactions of K21 inside the pocket of the targeted protein ( crystal structure of fibroblast collagenase-1 complexed to a diphenyl-ether sulphone based hydroxamic acid; PDB ID: 966C; Crystal structure of MMP-2 active site mutant in complex with APP-derived decapeptide inhibitor; PDB ID: 3AYU;Crystal structure of MMP8 complexed with hmr2909 PDB ID: 1BZS, Solution structure of SrtA from *Staphylococcus aureus (S. aureus)* in complex with 2-(aminomethyl)-3-hydroxy-4H-pyran-4-one based prodrug,, PDB ID: 6R1V; and Crystal structure of a hydroxamate based inhibitor EN140 in complex with the MMP-9 catalytic domain, PDB ID: 4WZV). The molecular docking studies of K21 were performed using Schrödinger Drug Discovery Suite Modelling Maestro 13.3 software. For MD studies, Desmond package was used. This study provides insights on molecular interactions between K21 and the target protein. The crystal structure of the protein was downloaded from Research Collaboratory for Structural Bioinformatics Protein Data Bank (RCSB PDB) (http://www.pdb.org). The in silico molecular docking studies were carried out sequentially as such: Ligand preparation, Protein preparation, and MD.

#### Preparation of ligands

The ligand, K21 was prepared using the ‘LigPrep’ tool in Schrödinger Drug Discovery Suite following the instructions described in the user manual. The structure of the ligand was sketched in Maestro using a 2D-sketcher. In the ‘LigPrep’ wizard, the possible states of ligand were generated using Epik algorithm, with the pH set to 7.2 ± 0.2. The output structures were saved as a maestro file.

##### Defining Binding (Active) site using Receptor Grid Generation

The prepared protein, as outlined in Sect. 2.1.1, was subsequently utilized to define the binding site. This crucial step was executed using the 'Receptor Grid Generation' tool from Schrödinger's suite. The procedure adhered to established protocols described in relevant scientific literature. Specifically, the process involved utilizing the centroid of the ligand in the co-crystallized protein–ligand complex. This centroid served as a reference point for determining the center of the grid. This approach ensures that the generated grid accurately encompasses the biologically relevant binding site, critical for the subsequent molecular docking simulations. By aligning the grid center with the ligand's centroid, the protocol enhances the precision of the docking predictions, ensuring that the simulated interactions occur within the experimentally observed binding region.

#### Ligand docking

The docking of prepared ligands into the receptor grid was conducted using the 'GLIDE' wizard, following the protocols detailed in the user manual, without further modifications to the prepared ligands. 'LigPrep' was used to prepare ligands. The chosen docking method was the Extra Precision (XP) mode within 'GLIDE', noted for its heightened accuracy in predicting ligand binding poses. The procedure adhered to the default settings of 'GLIDE', which included several key features: flexible ligand sampling to thoroughly explore the ligand's conformational possibilities, enabling the sampling of nitrogen inversion and ring conformations to consider alternative tautomeric and geometric states of the ligands and the incorporation of Epik state penalties into the docking scores. The inclusion of Epik state penalties is particularly significant as it accounts for the energetic costs associated with the protonation and tautomerization states of the ligands, thereby enhancing the predictive accuracy of the docking process.

#### Protein preparation

The protein crystal structure was prepared according to the guidelines provided in the user manual, utilizing the 'Protein Preparation' wizard in the Schrödinger Drug Discovery Suite and adhering to its default settings. The co-crystallized ligand/protein complex structure (PDB ID: 1BZS) was imported into Maestro from the Protein Data Bank (PDB). During the preparation process in Maestro, heteroatom states (HET states) were generated using the Epik tool, with the pH range set to 7.2 ± 0.2, reflecting physiological conditions. The protein's protonation states were optimized using PROPKA at a pH of 7.0. Additionally, water molecules located more than 3 Å from the ligand were removed to focus on the immediate solvation environment of the ligand–protein complex. Finally, the protein structure was minimized using the OPLS4 force field, ensuring a refined and stable conformation for subsequent analyses.

#### Molecular dynamics

The molecular dynamics (MD) simulations were meticulously executed using the Desmond package, part of the Schrödinger Drug Discovery Suite. The complex of the K21-receptor was initially embedded in an orthorhombic water box using the TIP3P water model. The dimensions of this box were carefully set to extend 10 Å in all three axes from any atom of the complex, ensuring ample space for molecular movement and interaction. Counterions, specifically sodium ions (Na +), were judiciously added to achieve charge neutrality in the system.

The MD simulations were conducted under the NPT ensemble, which maintains a constant number of particles (N), pressure (P), and temperature (T). The temperature was controlled at 300 K, and the pressure was set at 1.013 bar, mirroring standard atmospheric conditions. During the simulation, energy parameters were recorded at intervals of 40 ps, while the trajectory was captured every 10 ns, allowing for a detailed analysis of the system's dynamics over time.

A critical aspect of the simulations was using the OPLS-4 force field. This choice was driven by its ability to provide accurate and reliable interaction potentials, essential for the realistic modelling of molecular interactions. The stability and behaviour of the compounds in their dynamic states were monitored using several key metrics: root mean square deviation (RMSD), root mean square fluctuations (RMSF), and ligand contacts.

The computational aspect of this work was conducted on a robust Linux-x86_64 operating system, utilizing an Intel® Xeon® W-1270 CPU with a clock speed of 3.40 GHz. The simulations were accelerated using a GPU-Quadro RTX5000, equipped with NVIDIA version 470.129.06 drivers.

### In-Vivo micronucleus test of K21 In peripheral blood of CD-1 mice

The in-vivo micronucleus test of K21 in peripheral blood of CD-1 mice was conducted with standard operating procedures and in compliance with the OECD Principles of Good Practice [23], Good Laboratory Practices of the United States Food and Drug Administration [24] and ARRIVE guidelines. Characterization, identity, stability, and verification of the test item as received and tested were the accountability of the author’s team. This study evaluated whether the test item K21 caused cytogenetic damage, which results in the formation of micronuclei containing either lagging chromosome fragments or whole chromosomes. K21 (QAS Drug (with H2SO4, neutralized) Composition: K21 Quaternary Ammonium Silane-Functionalized (QAS) material in 100% Ethanol) Lot No: AP10-069 was generously supplied by Fitebac Technology / Largent Health.

For the positive control, an accurately weighed portion of Cyclophosphamide Monohydrate (CP; Sigma Aldrich, USA) was dissolved in 0.9% normal saline to obtain a final concentration of 5.0 mg/mL. An appropriate volume of K21 was diluted with an appropriate volume of ethanol. K21 concentration was calculated as follows: 0.502 g/g × 0.902 g/mL × 1000 mg/g = 452.8 mg/mL. Duplicate 200 μL samples of each test item formulation and vehicle control from the main tests were collected into 15 mL conical tubes on the day of preparation and diluted 50-fold or 9.80 mL in ethanol (100%) on the same day and stored at -10 to -25 °C until analysis.

The sample size for analysis of specimens will be derived by the following equation keeping the power of study equal to 90% and level of significance equal to 5%.

$$n= \frac{{\left({Z}_{1-\beta }+{Z}_{1-\frac{\alpha }{2}}\right)}^{2}\left( {{\sigma }_{1}}^{2}+ {{\sigma }_{2}}^{2}\right)}{{\left({\mu }_{1}- {\mu }_{2}\right)}^{2}}$$

$${Z}_{1-\beta }=\mathrm{Z score for power of study at }90\mathrm{\%}=1.28$$

$${Z}_{1-\frac{\alpha }{2}}=\mathrm{Z score for level of significance at }5\mathrm{\%}=1.96$$

$${\mu }_{1}-{\mu }_{2} (426.2- 423.2) =\mathrm{ mean difference }= 3$$

$${{\sigma }_{1}}^{2}+{{\sigma }_{2}}^{2}[(27.5)2 + (26.7)2] =\mathrm{ standard deviations of the groups }= 11.2$$

Sample size to be taken = 3 (Wei et al., 1998).

#### Housing and identification

Male and female mice were housed in separate Nalgene® cages, up to 3–5 mice per cage. The animal room atmosphere was stabilized (targeted ranges: temperature 18-26ºC, relative humidity 30–70%, greater than 10 air changes per hour) and supervised. The photo-cycle was 12 h light and 12 h dark. All animals were examined for general physical examination, fit to be admitted. The oral route of administration was selected as this is the intended route of human exposure (oral gavage). The test and control items were administered to animals via oral gavage using a blunt tip 20 G gavage needle inserted into the stomach of the animals. Each animal was dosed with a new sterile syringe (commercially available) and needle. Each group was sequentially dosed at intervals to ensure survival of the group (approximately 24-h intervals). Animals were closely observed for the first hour post-dose and then once daily for 4 days. Animals were euthanized (using exsanguination/cardiac perfusion with the use of injectable anesthetic agents (sodium pentobarbital) at the end of the four-day observation period. Main Study test and control item dosing formulations were administered to animals once as outlined in Table 2.

#### Blood sample collection and fixing

Blood samples (~ 0.5 mL) collected into K_2_EDTA were immediately transferred for further processing using the Litron MicroFlow® PLUS micronucleus analysis kit for mouse blood according to the manufacturer’s instructions. Each blood sample was mixed gently to ensure a homogeneous suspension and 100 μL of sample was removed and diluted with 350 μL Litron Anticoagulant/Diluent. The diluted blood (180 μL) was added to 2 mL of fixative (absolute methanol) kept at -70 to -90 °C at least overnight, vortexed/mixed briefly and stored for 6 to 7 days at -70 to -90 °C. Samples were washed out of fixative before being labelled for analysis of micronuclei by flow cytometry. Following vortexing, 12 mL ice-cold Buffer Solution was added immediately to each tube. Tubes were mixed by inversion and stored on ice until they were centrifuged at 351 × g for 5 min at 2–8 °C. The supernatant was removed, and the cell pellet resuspended in residual supernatant. Flow cytometry was performed using a MACS Quant Analyzer 10 in accordance with the Litron MicroFlow® PLUS micronucleus analysis kit manual. At least 2000 erythrocytes per animal (> 221,383) were counted to determine the proportion of RET, and at least 4000 RET per animal (10,000) were scored for the incidence of MN-RET.

For each sample, the %RET were calculated as follows:

1 $$\%RET = RET + MN-RET / RET + MN-RET + NCE + MN-NCE x 100$$

### Bacterial reverse mutation test of K21 molecule

Duplicate 200 μL samples of each K21 formulation and vehicle control from the main tests were collected into 15 mL conical tubes on the day of preparation and diluted 50-fold in ethanol (100%) on the same day and stored at -10 to -25 °C until analysis. *E. coli* strain WP2 *uvrA* was streaked on Nutrient Agar #2. The plates were incubated for 18–48 h at 37 ± 2ºC. Single colonies for each strain were streaked onto secondary master plates and incubated for 18–48 h at 37 ± 2ºC. Oxoid Nutrient Broth #2 (2.5%) (NB#2) was used for daily growth of tester strains. Top agar was composed of 0.6% BactoTM agar and 0.5% NaCl supplemented with 10 mL of 0.5 mM biotin solution and 0.2 mL of 25 mM HIS solution per 100 mL.

#### Metabolic activation

Since the bacteria do not have endogenous metabolic capacity, an exogenous metabolizing system was used, i.e., co-factor-supplemented post-mitochondrial fraction (S9) prepared from the livers of male Sprague–Dawley rats treated with an enzyme inducer (Aroclor-1254). S9 was supplied by Molecular Toxicology Inc. and was stored at -80 ± 10 °C. Immediately prior to use, S9 stock was thawed and an S9 mix was prepared containing the following components: 0.1 M NaH2PO4/Na2HPO4, pH 7.4, 5 mM Glucose-6-phosphate, 4 mM NADP, 33 mM KCl / 8 mM MgCl2, 10% rat liver S9. From the master plates, overnight cultures were inoculated and grown in NB#2 at 37 ± 2 °C with shaking at 210 rpm for 16 h. The overnight culture of *E. coli* strain WP2 *uvrA* was diluted in fresh NB#2 and incubated for 2.5 h to the desired density. The estimated density of the cultures ranged from 0.89 to 2.61 × 109 colony-forming units per mL as determined by spectrophotometric monitoring at 650 nm.

#### Treatment

In the preliminary and main study plate incorporation tests, 100 μL of the test item formulation was transferred into a sterile culture tube. S9 mix or 0.1 M phosphate buffer (pH 7.4) (0.5 mL) and 0.1 mL of an overnight culture (which corresponded to approximately 108 viable bacteria per tube) were added in an appropriate sequence. The molten top agar (2 mL) was added; the contents were gently vortexed and then layered on bottom agar (i.e., MGA for *S. typhimurium* strains, and SA1 agar for *E. coli*).

### Sortase a inhibition and biofilm formation assays

*Enterococcus. Faecalis (E. faecalis)* (ATCC 29212TM) was used to derive SrtA genes and purified through chromatography affinity of metal chelate with Ni-nitriloacetic acid (NTA) resin. A total of 100 μL was utilized as a total volume for each reaction with 120 mM NaCl, 50 mM Tris–HCL and 5 mM CaCl_2_ with a pH of 7.5. A total of 17 μg of purified SrtA with 250 ng of synthetic substrate (Dabcyl- LPETG-Edans) were added with a final concentration of 1% DMSO used as a solvent to each reaction mixture. All reactions were quantified using fluorometric intensity (350 nm excitation and 495 nm emission wavelengths) inside a microplate reader (FLx800, Bio Tek Instruments, Winooski, VT). For positive control, triphasiol was used as an SrtA inhibitor.

Single colony of *E. faecalis* from the agar plate (brain heart infusion broth) and cultured for 24 h in 2 ml of TSB medium (37 °C). The bacterial culture was diluted for 24 h using the TSB medium till OD_600_ reached up to 0.125. Triplicates of 1 ml aliquots were added in TSB medium (1:100) inside wells of 24-well plate with different concentrations of experimental solutions (20 µL). After static incubation for 18 h under 5% CO_2_ and 37 °C, the OD600 was measured again, medium aspirated and plates washed gently using 1 mL of sterile PBS (three times). After airdrying and staining for 1 h using 0.1% crystal violet, the plates were washed again thrice to remove excessive stain. The plates were dried and 200 µL of ethanol (95%) was added and OD_595_ measured using a microplate reader for the average values in triplicates.

### Statistical analysis

For SrtA inhibition and biofilm formation assays, statistical analysis was performed using SPSS software version 21.0 (IBM, Chicago, USA). Unless stated otherwise, statistical significance was set at *p* < *0.05* as all datasets were evaluated for homoscedasticity and normality. To evaluate if data sets were distributed normally, a one-way ANOVA and post-hoc comparison were performed.

## Results and discussion

**Fig. 4:**
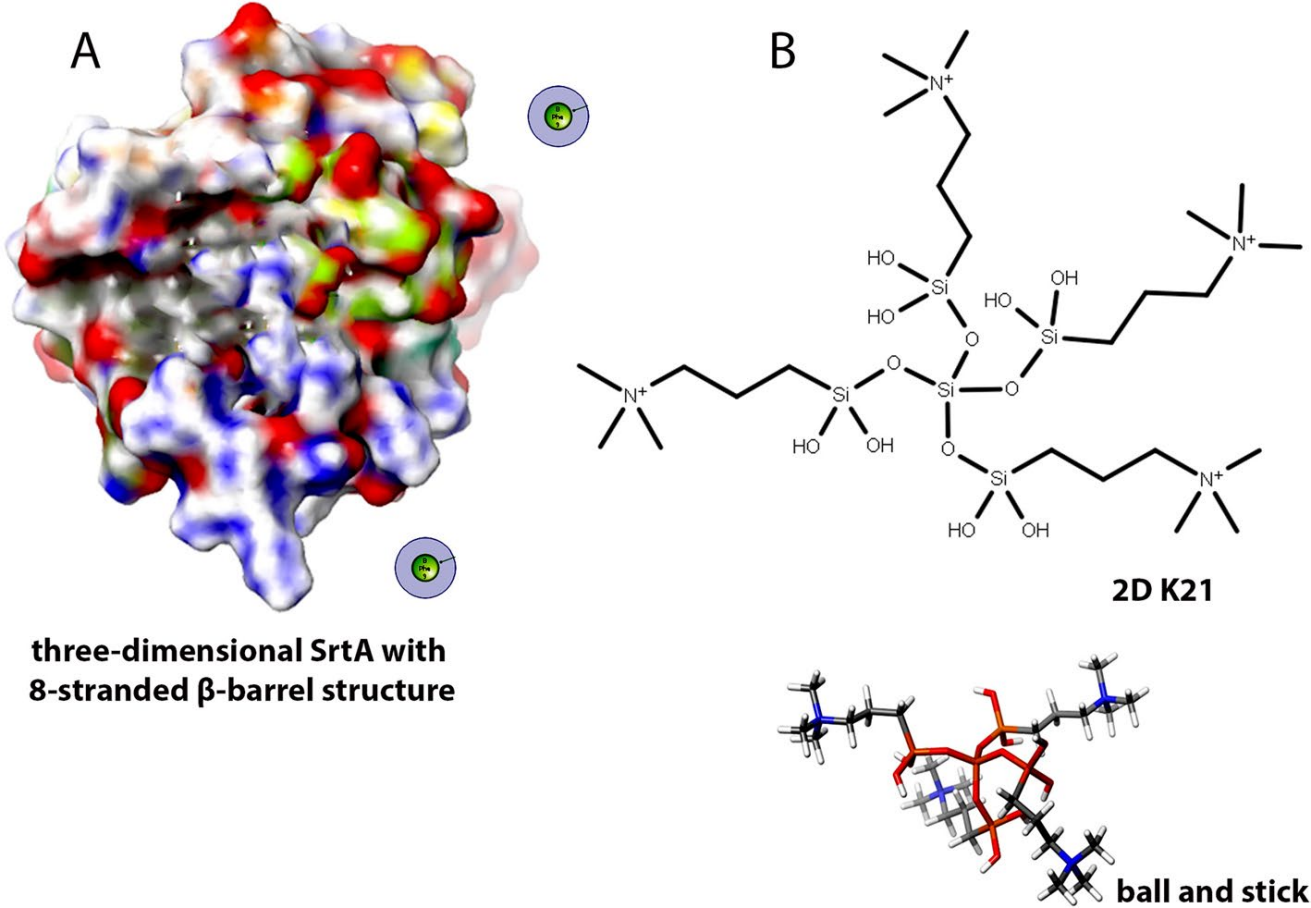
SrtA transpeptidase cleaving the LPXTG motifs linking penta-glycine from lipid II to the cleaved THR, containing 8-stranded β-barrel structure holding the catalytic pocket. Quaternary ammonium compounds or K21 is based on 1-octadecanaminium, N, N′-[[3,3-bis[[[3-(dimethyloctadecylammonio) propyl] dihydroxysilyl] oxy]-1,1,5,5-tetrahydroxy-1,5-trisiloxanediyl] di-3,1-propanediyl] bis [N, N-dimethyl-], chloride1:4 (C_92_H_2_0_4_Cl_4_N_4_O_12_Si_5_, using tetraethoxysilane and3-(triethoxysilyl)-propyl dimethyl octadecyl ammonium chloride

The MD simulation depicts that K21 forms a reasonably stable complex with the binding site of the targeted protein, 966C, 3AYU, 1BZS, 6R1V and 4WZV (Fig. 2). Figure 4 depicts the results from MD simulations that include RMSD of the complex (K21 in the protein), the amino acids, and the critical atoms of the compounds implied in the binding.

### Molecular dynamics simulation between k21 and proteins

As depicted in Fig. 4, the first column shows the protein–ligand complex’ RMSD (plot for the 10 ns trajectory. The RMSD values of the protein are on (left y-axis) and the ligand on (K21; right y-axis). The RMSD changes of 1–3 Å units are considered stable. The schematic of detailed atom interaction(s) with protein residue(s) for more than 30% of the simulation time is shown as follows: blue, water bridges; green, hydrogen bond; pink, ionic bond; and purple, hydrophobic interactions. The fraction (y-axis) represents the percentage of interaction in the 100 ns trajectory. For instance, 0.7 equals to 70% interaction in the simulation. The key amino acid interactions with the K21 for each protein is shown.

Protein–ligand interactions are classified into Hydrogen Bonds, Hydrophobic, Ionic and Water Bridges. Each of these has certain subtypes, that can be investigated through the 'Simulation Interactions Diagram' panel as shown in the Fig. 4. Protein Ligand root-mean-square deviation to indicate simulation time and structural stability.

$$RMS{D}_{{\text{x}}}=\sqrt{\frac{1}{N}\sum_{i=1}^{N}\left({r{\prime}}_{{\text{i}}}\left({t}_{{\text{x}}}\right)\right)-{r}_{{\text{i}}}\left({t}_{ref}\right){)}^{2}}$$

The RMSD for frame x is: where N was the number of atoms in the atom selection; t_*ref*_ was the reference time, (typically the first frame was used as the reference and it was regarded as time t = 0); and r' was the position of the selected atoms in frame x after superimposing on the reference frame, where frame x was recorded at time TX. The procedure was repeated for every frame in the simulation trajectory. Simulation time for all the MMPs (1, 2, 8 and 9) and SrtA-A were 10 nsec. The plots in Fig. 1 demonstrated the RMSD evolution of the Proteins (MMP1,2,8 and SrtA: left Y-axis). The RMSD changes of 1–3 Å units are considered acceptable.

The protein and ligand RMSD of 1–3 Å is considered stable [11]. The MMP1-K21 complex results showed that the protein RMSD is within 3 Å whereas the ligand RMSD is more than stable range. The interaction shows that K21 formed high interaction (water bridges) with aspartic acid (ASP 200) followed by hydrogen bond with ASP 175. The MMP2-K21 complex showed that the protein RMSD range is within 0 – 2.8 Å, whereas the ligand RMSD was 0 – 13.5 Å. The trajectory shows that K21 mainly forms hydrogen bond interaction with ASP 44, ASP 154, alanine (ALA 36) and isoleucine (ILE 155); water bridges with ASP 44, valine (VAL 41) and Threonine THR 46. The MMP8-K21 complex showed that the RMSD of the protein is within the range of 0.25–2.25 Å whereas the ligand RMSD is 0–24 Å. K21 was found to mainly form hydrogen bond, ionic bond, and water bridges interaction with aspartic ASP 232. The SrtA -K21 complex (Fig. 4) showed that both the RMSD of the protein and ligand fell out of the stable range at 0–3.2 Å and 0–16 Å respectively. K21 was found to form hydrogen bond, ionic bond, and water bridges interaction with ASP 118; hydrogen bond and water bridges with glutamine (GLN 55) and Glutathione (GLU 146). The MMP9-K21 complex showed that the RMSD of the protein is within the range of 0–2.4 Å, while the ligand RMSD fluctuates in the range of 0 – 13.5 Å. K21 was found to form mainly ionic bond with HIS 226, HIS 230 and HIS 236, and hydrogen bonds with proline (PRO 246) and tyrosine (TYR 248) (Table 1). The results suggest that K21 forms multiple interactions with strong bonds with 3AYU.

**Table 1:** The interactions of K21 with the key amino acid, type of interaction and bond length

MMP/PDB ID	2D interaction diagram between Protein and Ligand	Key Amino Acid(s)	Interaction type	Bond length, Å
**MMP1/ 996C**		ASP 200	Water-mediated hydrogen bond	1.50, 2.40
		ASP 200	Water-mediated hydrogen bond	1.50, 1.93
**MMP2/3AYU**		ASP 154	Hydrogen bond	1.62
		THR 46	Hydrogen bond	1.57
		ASP 44	Hydrogen bond	1.68
		ASP 44	Hydrogen bond	1.69
		ILE 155	Hydrogen bond	1.61
**MMP8/1BZS**		ASP235	Hydrogen bond	2.02
**MMP9/4WZV**		TYR 248	Hydrogen bond	1.56
**SrtA/6R1V**		GLN 55	Hydrogen bond	1.80
		ASP 188	Hydrogen bond	1.53, 1.91

In the context of the RMSD graphs shown in Fig. 3 for the protein–ligand complexes, particularly in the study involving the K-21 ligand, the observed lack of equilibrium can be primarily attributed to the unique characteristics of the ligand itself. The K-21 ligand is characterized by its long quaternary alkyl chains, which exhibit significant conformational flexibility. This high flexibility leads to many potential conformations for the ligand, adding complexity to the molecular dynamics (MD) simulation conducted using Desmond in Schrödinger. Such a wide range of conformations requires extensive computational resources to explore and stabilize within the simulation timeframe accurately. Despite this complexity, the MD simulation results reflected in the RMSD graphs do not show extreme or erratic fluctuations in the ligand–protein complex. Rather, they reveal a steady evolution of the complex's conformation over time, suggesting a consistent interaction pattern between the ligand and the protein despite its high conformational variability. This steady conformational evolution is critical in understanding the dynamic nature of ligand–protein interactions in systems with significant conformational flexibility.

### MMP1-K21 interaction

In the MMP1 (966C)-K21 complex, K21 has sporadically interacted with ASP200. The MD simulation did not show strong interactions between MMP1 and K21in the 10 ns trajectory.

### MMP2 – K21 interaction

In the MMP2 (3AYU)-K21 complex, K21 has interacted the most with ASP 44 as shown by the intense and dark orange blocks. K21 also interacted with tryptophan 42, VAL41, and ILE 155 over the trajectory of 10 ns.

### MMP8 – K21 interaction

The interactions between MMP8 (1BZS) and K21 over the trajectory of 10 ns was captured. The report showed strong interaction between K21 with serin 209 for the first four nanosecond only, then with ASP232 from 4 to 10 ns.

### MMP9 – K21 interaction

In the MMP9 (4WZV)-K21 complex, K21 has interacted the most with HIS226 as shown by moderately intense orange blocks. K21 also consistently interacted with HIS236, HIS 230, and phenylalanine 250 over the course of 10 ns. Another two notable interactions were with PRO 246, from 0 to 6.5 ns mark; and moderately intense but sporadic interaction with TYR248 over the 10 ns.

### SrtA – K21 interaction

The SrtA (6R1V) formed strong interactions with K21 over the trajectory of 10 ns. Intense dark orange bock was observed with ASP118, followed by glutamic acid 146 and glycine 55. K21 also consistently formed interaction with lysine 119. The protein-K21 complexes were found to be stable over the 10 ns trajectory. We postulate that K21 deters the formation of collagen complexes with the MMPs and SrtA. The centroid of K21, consisting of ammonium silane core, sits in the active site of the protein. On the other hand, the four long saturated alkyl chain containing 18 carbons each, possesses the flexibility for the carbon backbone to fold and move around the protein, thereby deterring other formation or interaction between MMP or SrtA with collagen.

### Target pocket binding of MMPs and SrtA

**Fig. 5:**
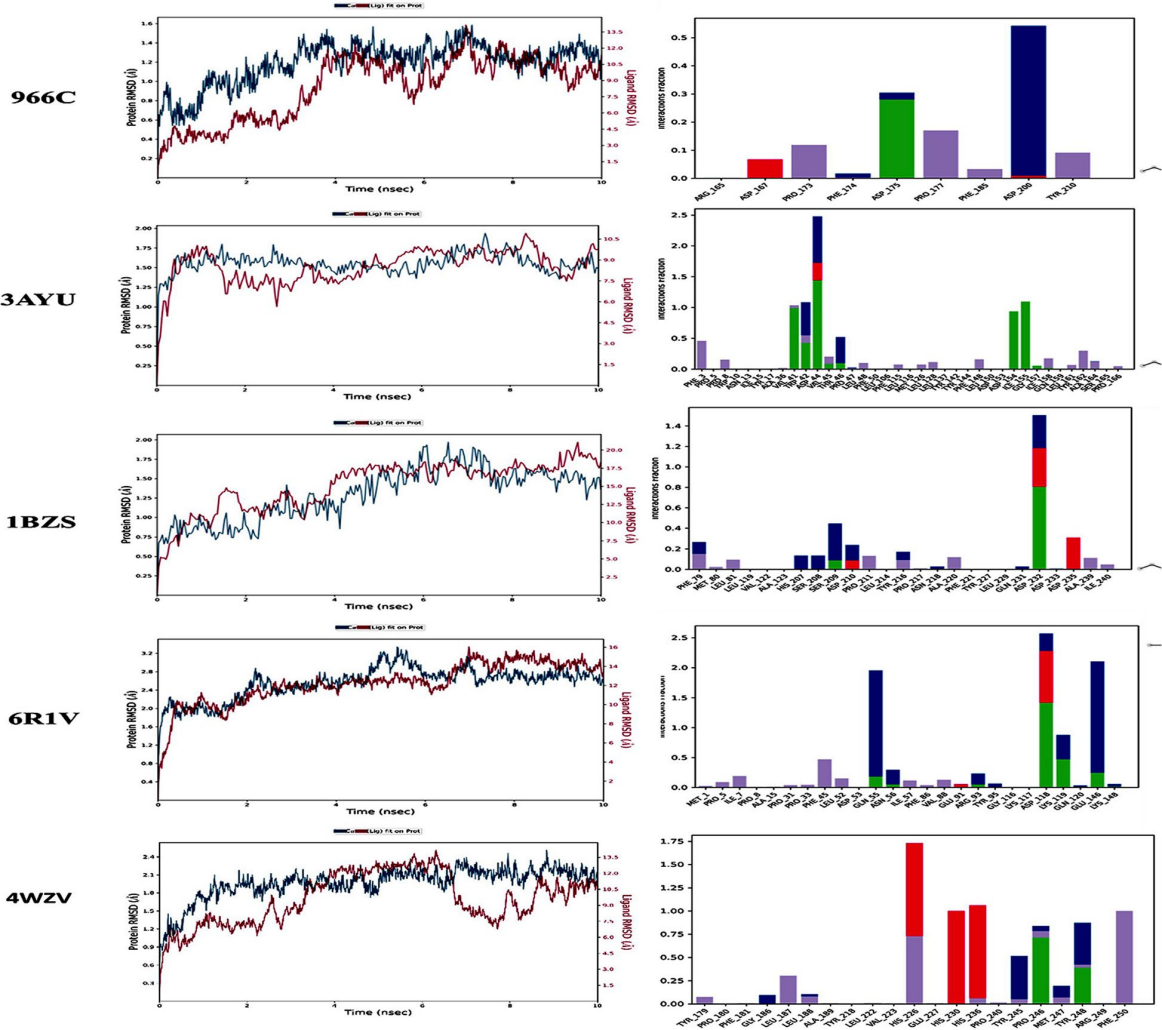
Molecular dynamics (MD) simulation (10 ns) of K21 with MMP1 (PDB ID: 966C), MMP2 (PDB ID: 3AYU), MMP8 (PDB ID: 1BZS), Sortase A (PDB ID: 6R1V) and MMP9 (PDB ID: 4WZV)

The binding pockets of MMPs are well-reported in scientific literature [25]. The MD of K21 with the MMPs were simulated for a trajectory of 10 ns. The interaction types, interactions with key amino acid residues and interaction intensity were recorded. Based on the reported MMPs pocket in the literature [26], the pocket(s) of interaction between K21 and the respective MMPs in this MD simulation were elucidated and proposed in Fig. 5.

**Fig. 6:**
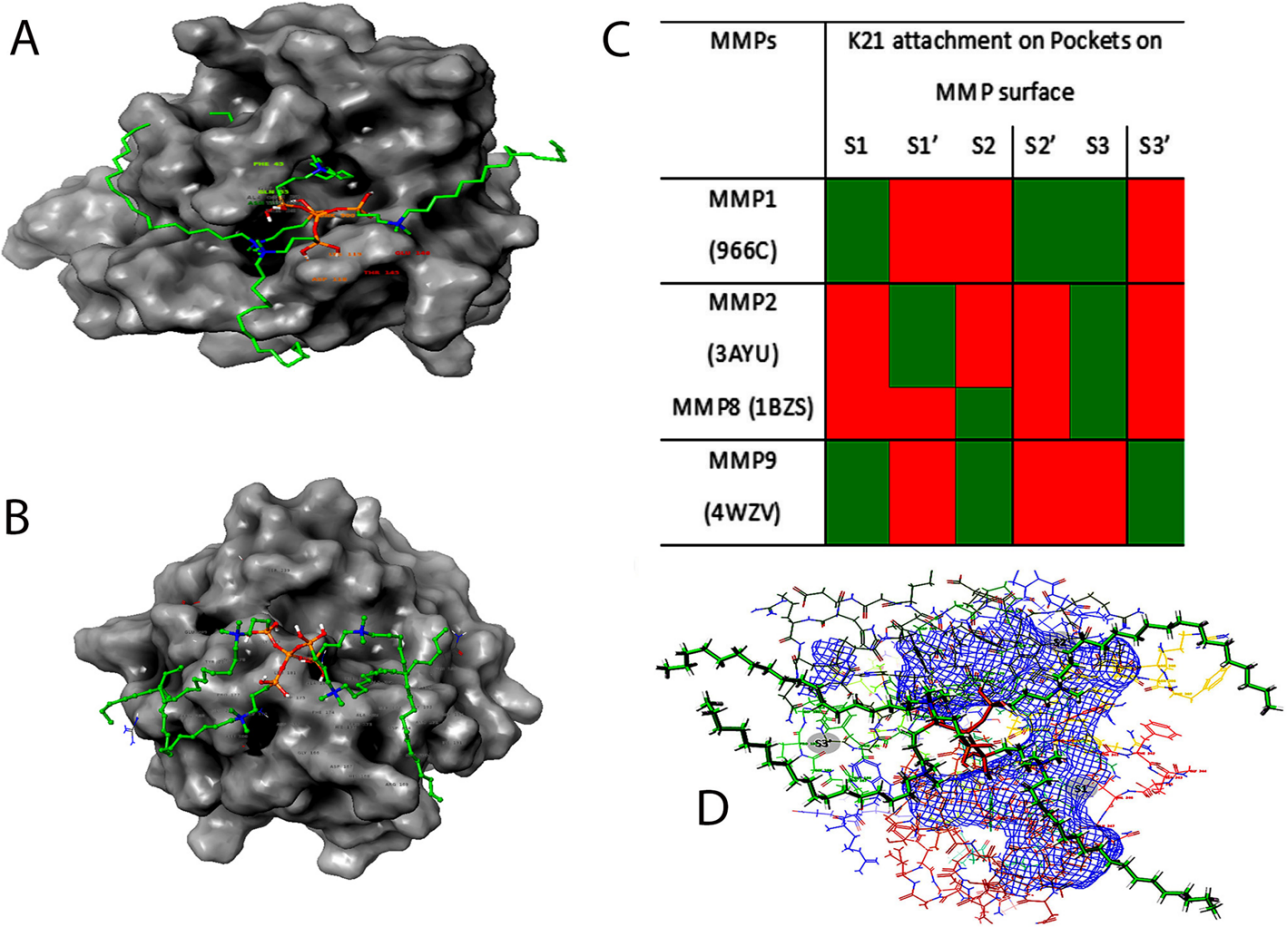
**A** The quaternary ammonium silane attaches at specific pockets on MMPs producing a “Clouting motion effect” preventing the attachment of other ligands. **B** The arrows indicate attachment of K21 chain on various clefts on MMP surfaces. The QAS molecule depicts a smacking by this attachment all over the MMP active sites preventing other molecules to ascribe on MMP structure. **D** 3D interactions depicting Clouting Motion Effect of K21 with MMPs

As MMP's are accountable for the turnover of ECM proteins, their exaggerated manifestation and activation plays a main role in the pathogenesis of various oral diseases [27]. In the MMP structure, the catalytic domain possesses an increased degree of structural similarity. The active site is a shallow groove like region located in the horizontal direction (Fig. 6A, B) having unprimed pockets on the left half of catalytic zinc ions (Pockets S1, S2 and S3) while primed pockets are present on the right side of catalytic zinc ions (Pockets S1’, S2; and S3’) [28]. Thus, the active catalytic cleft has six pockets as binding regions from S1 to S3 and S1’ to S3’ (Fig. 6C). Pockets of S1’ region of various MMPs are more pronounced, less solvent exposed and are of variable sizes making them as target regions for attachment by MMP inhibitors [29]. The differences in the sizes of these pockets are attributed to the varying amino acid residues with smaller amino acids responsible for developing a large pocket and vice versa.

MD in relation to MMPs is the mechanism of interaction between molecules and atoms of specific region of MMPs and its inhibitors or substrates. MD simulations provide vital information on the process of MMPs binding onto ECM substrates like elastin and collagen. This helps in assessing the molecular mechanism of structural determinants and specific substrates that govern their binding. MD explains the action of MMPs cleaving the proteins of ECM by elucidating catalytic action, active sites alterations and orientation of metal ions required for its actions. Binding of MMP inhibitors on specific MMPs catalytic sites provides insights into antiMMP action. This impacts the overall turnover of the ECM altering the homeostasis. MD also depicts allosteric regulation by displaying binding of substrate at distinct site away from catalytic sites affecting the MMP activity. This leads to structural alteration and affects the proteolytic action of MMPs [17, 30].

**Fig. 7:**
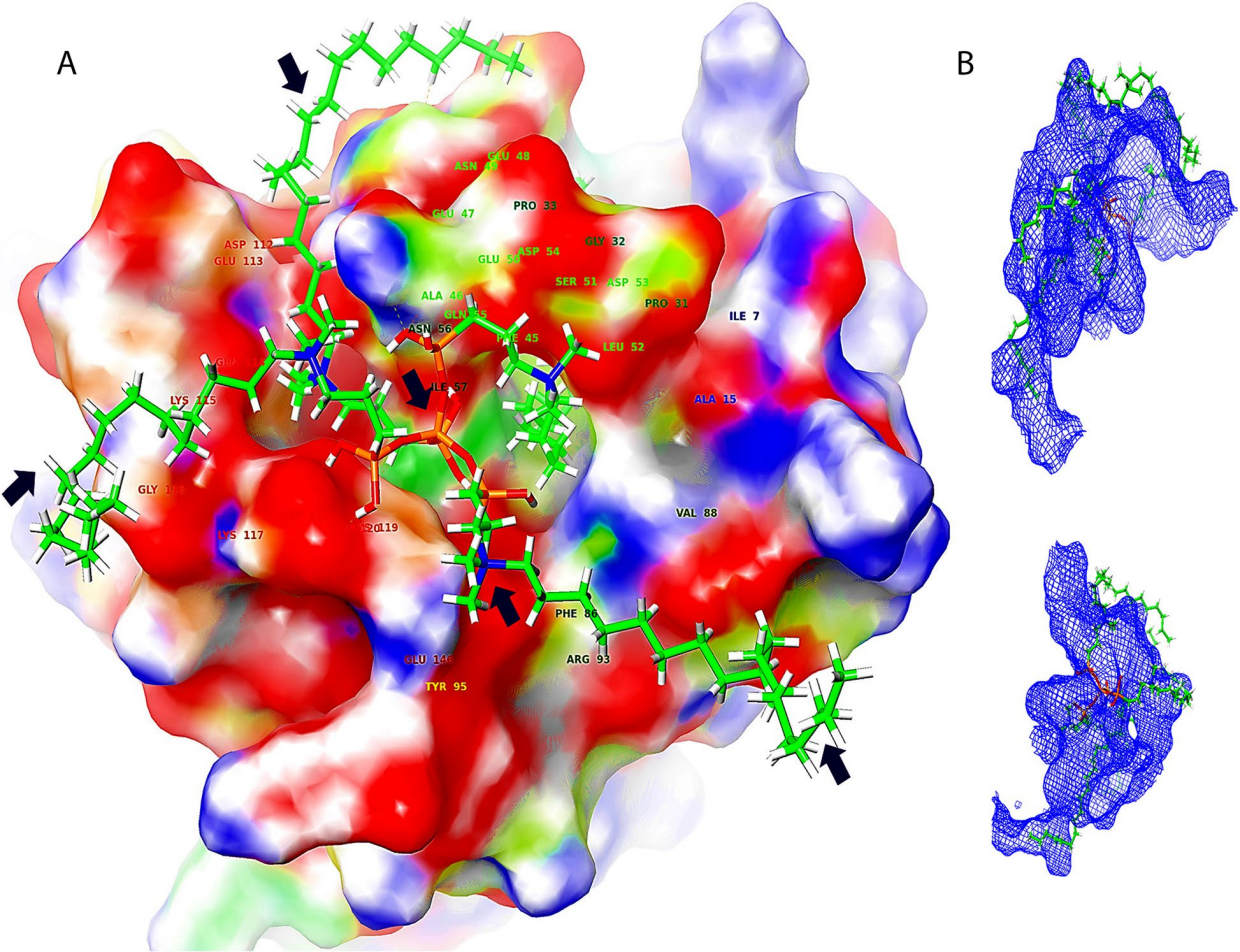
“Clouting Motion Effect” of K21 on Sortase A comparable to the effect found on MMPs. Arrows depict attachment of K21 on SrtA sites preventing the action of SrtA. Mesh appearance of the same

MMP8 attaches collagen fibrils at a certain peptide bond and breaks the collagen molecule into one-fourth and three-forth fragments [31]. As the peptide lose its triple helix structure, attack by MMP2 and MMP9 gelatinases further deteriorate the structure [32]. As per the three-dimensional (3D) image analysis (Fig S1), K21 binds onto most active sites of unprimed and primed pockets of MMPs and exerts a *“clouting motion effect”* covering a larger attached portion on MMPs preventing attachment of other substrates (Fig. 6A, B, D). “*Clouting motion effect*” eventually prevents the attachment of other molecules to their potential binding sites, drastically reducing the MMP potential (Fig. 7). This could be the reason for its antiprotease activity. The molecular simulation depicted that K21 binds to the amino acid residues of S1 pocket of MMP1 (S1) and MMP9 whereas S1’ pocket of MMP2 (S1). The hydroxyl groups of K21 occupied the S1’ pocket of MMP1 lined with hydrophobic amino acid residues namely ASP 154, THR 46, ASP 44 and ILE 155. Also, K21 formed a strong water mediated hydrogen bond with S1 pocket of amino acid residues of ASP 200 of MMP1 and ionic bonding with S1’ pocket amino acid residues of HIS 226 and HIS 230 of MMP9. This specific binding of K21 on S1 and S1’ pockets may alter the arrangement of the active site of MMPs, making them unable to bind the complementary peptide sequence for collagen. The K21-MMP complex can prevent the hydrolysis of peptide bonds of collagen by MMPs, restoring the fibril integrity within the dentin layer [19]. The selective binding of K21 on the active sites of MMPs prevents it attachment on collagen fibers [33]. This suggests that K21 can prevent the MMP mediated lysis of vulnerable collagen fibrils. Thus, “*clouting effect*” of K21 might prove to be a potent MMP inhibitor preventing periodontal diseases that involve collagen degradation (Fig S1). Previously, studies suggesting that the S1’s loop flexibility may play an important role in the binding process have been performed by Cesar et al. The configurations indicate the tunnel-like binding pocket which is well defined corresponding to the most relevant and representative configurations.

Collagen fibril structure remains unchanged with intact cross banding after treatment with K21 [34]. The authors may also speculate that the silicon ion within the K21 (silane) structure serves as a nidus for nucleation of apatite crystals. Thus, K21 maintains collagen stability due to creation of siloxane chains influencing the mechanical properties. It was also observed that K21 attached onto the S2 pockets on MMP8 (S1) and MMP9 (S1) and S3 pockets of MMP1, MMP2, MMP8 and S3’ pocket of MMP9. The novelty of this study is that it has displayed the molecular mechanism of action highlighting *Clouting effect* of four quaternary arms of QAS on MMPs. Occupying multiple target pockets at different time points of interaction shows that K21 provides sustainable action of MMP inhibition.

Bacteria exhibit diverse proteins on cell surface that assist them to efficiently interact with their environment. Gram-positive bacteria like *Streptococcus mutans (S. mutans)* utilize SrtA enzyme to covalently bind proteins to the cell wall. SrtA also serve as virulence factor and play a key role in nutrient procurement, promoting bacterial adhesion and inhibition of immune response [35]. Thus, a substantial amount of research is under process to explain the method of SrtA-facilitated catalysis and to understand SrtA inhibition pathway. The mechanism regarding biofilm development remains unclear, studies have shown that the biofilm formation and progress can be inhibited by targeting and bringing about conformational change within the SrtA molecule affecting the attachment phase [36]. The inhibitory potency of the compounds was calculated and presented as IC50 values as depicted in Table 1. The biofilm formation or multiplication was significantly reduced with the use of K21 molecules compared to the control biofilms. SrtA inhibition can weaken or prevent biofilm formation resulting in reduction of virulence factor decreasing the pathogenicity. This will enhance susceptibility to immune clearance and antibacterial treatment. MD of SrtA attachments provides new insights of developing antibiotics targeting surface proteins to decrease biofilm formation as compared to the conventional treatment of targeting cell wall or protein synthesis. This furnishes solutions to treat infections due to antibiotic resistant bacteria (MRSA). Thus, SrtA could serve as a potential target to eliminate specific bacteria and can reduce the chances of off- target impact caused by broad spectrum antibiotics [37, 38]. SrtA cleaves the sorting signal between the substrate threonine and glycine, the LPXTG motif, covalently attaching the pathogenic bacteria to the host cell wall (Mazmanian et al., Liu et al.). Based on these previous studies, SrtA has been evaluated to primarily contribute to the substrate catalysis and binding, the LPXTG motif. This indicates that due to the inhibitor binding, SrtA cannot bind with the substrate, the LPXTG motif, leading to the loss of catalytic activity.

The polar capacity of K21 allowed it to produce a charge- charge connection that can attach onto to the binding pockets of SrtA surfaces. The crystal arrangement of SrtA has eight stranded folds with multiple loops and two small helices having β7 and β8 strands making a hydrophobic strand with amino acids. Surface adhesins of *S. mutans* are attached to the cell wall by SrtA transpeptidase that covalently bonds with LPXTG motif comprehending surface proteins with the cell wall envelope of Gram-positive bacteria [39]. The strong bonding of K21 with SrtA impedes the catalytic action causing decreased anchorage of the surface proteins. SrtA possesses 206 amino acids with C-terminal catalytic domain and N-terminal membrane-spanning region. SrtA facilitated ligation was used for C-terminal protein modification due to higher specificity and productivity. The secondary structural elements like α helices and β strands fluctuated less compared to the tails (C-terminal and N-terminal) of the SrtA protein. These conformational transformations were observed due to specific interaction of K21 with SrtA protein (Daood et al.).

**Fig. 8:**
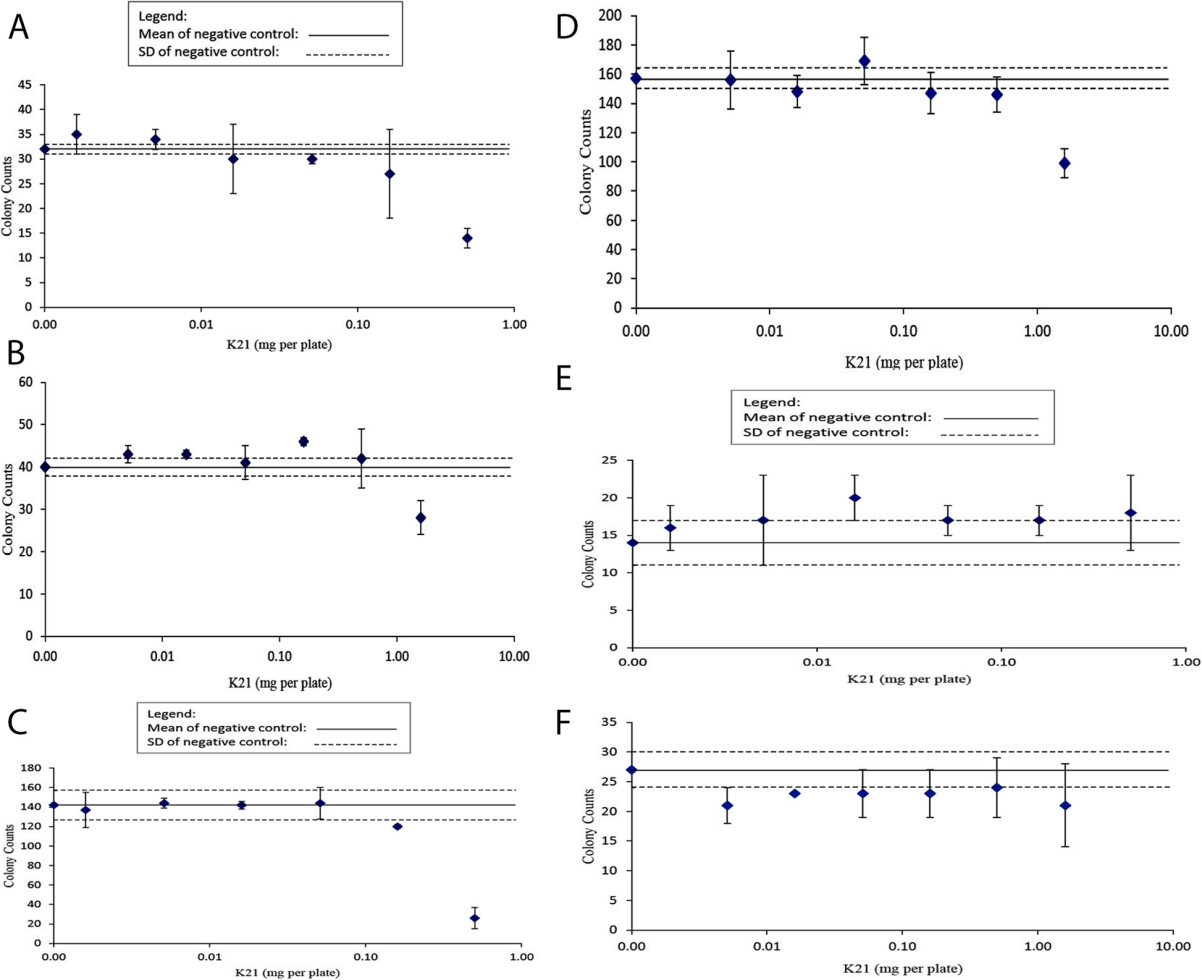
**A** (TA98, -S9)—Mean colony counts ± SD were plotted against the test item concentration; **B** (TA98, + S9)—Mean colony counts ± SD were plotted against the test item concentration.; **C** (TA100, -S9)—Mean colony counts ± SD were plotted against the test item concentration; **D** (TA100, + S9)—Mean colony counts ± SD were plotted against the test item concentration; **E** (TA1535, -S9)—Mean colony counts ± SD were plotted against the test item concentration; **F** (TA1535, + S9)—Mean colony counts ± SD were plotted against the test item concentration

Cytotoxicity, as indicated by a reduction in colony counts and/or the background bacterial lawn, was observed for all *E Coli* strains in the absence of S9 at the highest concentration evaluated (0.50 mg/plate). In addition, cytotoxicity was observed for TA1537 at the second highest concentration of 0.16 mg/plate. Clear cytotoxicity was not observed for any *Salmonella* strain in the presence of S96 or WP2 *uvrA* in the absence or presence of S9 (Table 2). Following treatment with K21, no increase in the mean number of revertant counts was ≥ 3.0 (TA1535 and TA1537) or ≥ 2.0 (TA98, TA100, and WP2 *uvrA*) times the mean concurrent negative control (Table 2). The mean ± SD colony counts per plate for all concentrations of K21 overlapped or fell below the concurrent negative control range (mean ± SD) for all tester strains and conditions (Fig. 8) with the following exception. At 0.16 mg/plate, TA98 in the presence of S9 had an increase in colony counts (46 ± 1) that was slightly greater than the negative control (40 ± 2) (Fig. 8). The apparent increase in revertant colonies was attributed to the low variability of the concurrent negative control. Consequently, as this exception was < 2.0 times the mean concurrent negative control (Table 2) and occurred in the absence of cytotoxicity, with no evidence of a concentration dependency, it was considered as consistent with a negative response. Therefore, K21 was negative, i.e., did not induce reverse mutations, using the plate incorporation method.

**Table 2:** Results for main study plate incorporation assay

K21 mg per plate	Colony Counts			Mean ± SD	Fold	*p* value	Mean ± SD of Transformed	Lawn	Precipitate
TA98, -S9									
0	31	32	33	32 ± 1	1.0		5.66 ± 0.09	NL	NP
0.0016	31	36	38	35 ± 4	1.1		5.91 ± 0.31	NL	NP
0.0051	32	34	35	34 ± 2	1.1		5.80 ± 0.13	NL	NP
0.016	24	28	38	30 ± 7	0.9		5.45 ± 0.65	NL	NP
0.051	29	30	31	30 ± 1	0.9		5.48 ± 0.09	NL	NP
0.16	17	30	33	27 ± 9	0.8		5.11 ± 0.87	NL	NP
0.50	12	14	16	14 ± 2	0.4		3.74 ± 0.27	ER	SP
2-NF, 5 µg	3212	3605	3809	3542 ± 303	110.7	< 0.0001	59.48 ± 2.57	NL	NP
TA98, + S9									
0	38	40	41	40 ± 2	1.0		6.30 ± 01.2	NL	NP
0.0051	41	44	45	43 ± 2	1.1		6.58 ± 0.16	NL	NP
0.016	43	43	44	43 ± 1	1.1		6.58 ± 0.04	NL	NP
0.051	37	42	45	41 ± 4	1.0		6.42 ± 0.32	NL	NP
0.16	45	46	47	46 ± 1	1.2		6.78 ± 0.07	NL	NP
0.50	36	39	50	42 ± 7	1.1		6.44 ± 0.56	NL	SP
1.6	24	28	31	28 ± 4	0.7		5.25 ± 0.34	*	MP
B[a]P, 5 μg	520	553	603	559 ± 42	14.0	< 0.0001	23.63 ± 0.88	NL	NL
**TA100, -S9**									
0	131	135	159	142 ± 15	1.0		11.89 ± 0.63	NL	NP
0.0016	119	139	154	137 ± 18	1.0		11.70 ± 0.75	NL	NP
0.0051	139	145	148	144 ± 5	1.0		12.00 ± 0.19	NL	NP
0.016	139	140	147	142 ± 4	1.0		11.92 ± 0.18	NL	NP
0.051	125	151	155	144 ± 16	1.0		11.97 ± 0.69	NL	NP
0.16	118	119	122	120 ± 2	0.8		10.94 ± 0.09	NL	NP
0.50	17	24	38	26 ± 11	0.2		5.06 ± 1.03	ER	SP
Na Az, 5 μg	1986	1849	1857	1897 ± 77	13.4	< 0.0001	43.55 ± 0.88	NL	NP
**TA100, + S9**									
0	153	153	165	157 ± 7	1.0		12.53 ± 0.27	NL	NP
0.0051	134	161	174	156 ± 20	1.0		12.49 ± 0.83	NL	NP
0.016	136	152	156	148 ± 11	0.9		12.16 ± 0.44	NL	NP
0.051	150	178	178	169 ± 16	1.1		12.98 ± 0.63	NL	NP
0.16	133	148	160	147 ± 14	0.9		12.12 ± 0.56	NL	NP
0.50	139	139	159	146 ± 12	0.9		12.06 ± 0.47	NL	SP
1.6	87	104	106	99 ± 10	0.6		9.94 ± 0.53	*	MP
B[a]P, 5 μg	1508	1500	1403	1470 ± 58	9.4	< 0.0001	38.34 ± 0.77	NL	NP

*B[a]P* Benzo[a]pyrene, *NaAz* Sodium azide, *2-NF* 2-Nitrofluorene, *Transformed* Square root of colony counts, *Fold* Mean colony counts ÷ Mean colony counts of negative control, *NL* Normal, *SR* Slight Reduction, *MR* Moderate Reduction, *ER* Extreme Reduction, AB: Absence, *NP* None, *SP* Slight, *MP* Moderate, *EP* Extreme

*p*-values calculated if Fold ≥ 3.0 (TA1535 and

TA1537) or ≥ 2.0 (TA98, TA100, and

WP2 *uvrA*)

Background Lawn Toxicity:

^*^: Lawn not scorable due to precipitate

Test Item Precipitate:

Colony Counts Mean ± SD Fold Lawn Precipitate

Mean ± SD

The Relative (Rel.) %RET for K21 at 500, 1000 and 2000 mg/kg at 36–45 h was 96.3%, 116.1%, and 81.6% for the males and 117.4%, 105.0%, and 95.7% for the females, respectively (Supplementary Material 1). At 60–72 h, the Rel. %RET for K21 at 2000 mg/kg was 155.0% and 70.4% for males and females, respectively. The Rel. %RET for the positive control, CP, was 37.4% and 67.2% for males and females, respectively. Therefore, all treatment groups produced valid results and the test item was evaluated up to the maximum recommended concentration of 2000 mg/kg. The mean %MN-RET for K21 at 500, 1000 and 2000 mg/kg was 0.17%, 0.25% and 0.23% for males and 0.21%, 0.24% and 0.21% for females at 36–45 h. K21 was evaluated up to the maximum recommended dose of 2000 mg/kg. The negative/vehicle control (ethanol 100%), K21, and positive control (CP) dosing formulations were administered to animals once by oral gavage. There were no abnormal clinical findings observed in any of the animals during the Dose Range Finding or Main Study. Following K21 treatment, no statistically significant increases in %MN-RET were observed, and all results were within the normal characteristic distribution of the historical negative control data. When evaluated up to 2000 mg/kg, K21 was negative for micronuclei formation in the peripheral blood of mice.

Results obtained of Molecular dynamic interactions between QAS/K21, MMPs and SrtA can be used to develop a novel biomaterial for SrtA and MMP inhibitors. These in-silico dynamics can provide the road map for use of this novel formulation in preventing collagenolytic action of MMPs and possible preservation of periodontal health. MD has also shown that QAS also causes conformational changes in SrtA structure, thereby showcasing antibacterial action and can serve as cavity disinfectant preventing secondary caries under restoration [10]. MMP inhibitors and QAS can be integrated into drug delivery systems for targeted delivery to oral tissues [8]. Knowing the molecular dynamics of these interactions can assist in designing effective drug delivery systems for the management of various dental diseases [29, 40]. The growing demand for innovative antimicrobials to treat life-threatening diseases caused by the global expansion of multidrug-resistant bacterial pathogens contrasts sharply with present investment in their development, notably in the field of synthetic small molecules. Our work proposes a strategy plan to significantly increase our ability to identify and develop novel antimicrobials, therefore fueling the translational pipeline for future generations. The fact that the biological outcome is directly dependent on known toxicity processes (micronuclei formation) is favorable since it improves the efficacy of the resultant prediction algorithms. For some endpoints, the intricacy of chemically induced toxicity processes and pathogenesis, along with an understanding of simulated pathways, facilitates the development of standardized tests and the creation of homogenous datasets. However, biological mechanisms, like those driving other toxicities, are challenging to include into computer models. Moreover, the authors do acknowledge the limitations associated with the study. With the use of molecular simulation and dynamics, there is always a lack of confidence in the ability to record accurate binding energies. Therefore, experimental validation via genotoxicity and micronuclei evaluation were validated. As force fields may use different considerations and simplifications, the description of the system may be inaccurate. In addition, primary limitation of the computational study is the potential inadequacy of the simulation timeframe in fully capturing the dynamic behaviour of the K-21 ligand–protein complex, particularly considering the high conformational flexibility of the K-21 ligand. The simulation duration might not be sufficient to observe all relevant conformational states, including transient or rare states crucial for a comprehensive understanding of the ligand–protein interactions in systems with significant conformational variability. While they provide valuable insights, the results are essentially predictions of how K-21 will interact with the protein in a solvated environment and should be interpreted with an understanding of these constraints.

## Conclusion

Molecular Simulation depicted that K21 has a specific pocket binding on various MMPs and SrtA surfaces producing a classical clouting effect inhibiting the catalytic action of MMPs and SrtA enzymes. The K21 molecule did not induce micronuclei, and the test item was not mutagenic to *S. typhimurium* strains and *E. coli* in the absence and presence of metabolic activation when tested up to the limit of cytotoxicity or solubility.

### Supplementary Information

**Supplementary Material 1.**

**Supplementary Material 2.**

## Data Availability

The datasets used and/or analysed during the current study are available  from the corresponding author on reasonable request.

## Abbreviations

AL: Alanine
ASP: Aspartic acid
E. faecalis: Enterococcus. Faecalis
ELISA: Enzyme linked Immuno Assay
ECM: Extracellular matrix
GLN: Glutamine
GLU: Glutathione
HIS: Histidine
ILE: Isoleucine
MD: Molecular dynamics
ns: Nano seconds
PRO: Proline
QAS codenamed as k21: Quaternary Ammonium Silane
RCSB PDB: Research Collaboratory for Structural Bioinformatics Protein Data Bank
RMSD: Root means square deviation
RMSF: Root means square fluctuations
S. typhimurium: Salmonella typhimurium
SrtA: Sortase A
S. aureus: Staphylococcus aureus
S. mutans: Streptococcus mutans
3D: Three-Dimensional
TIMPs: Tissue inhibitors of metalloproteinases
THR: Threonine
TYR: Tyrosine
VAL: Valine
