# Supplementary material for: Newly discovered clouting interplay between matrix metalloproteinases structures and novel quaternary Ammonium K21: computational and in-vivo testing
Source: BMC Oral Health. 2024 Mar 25;24:382. doi: 10.1186/s12903-024-04069-0 (PMC10964527; doi:10.1186/s12903-024-04069-0)
Supplement: Supplementary file 1 — Supplementary Material 1. [file 12903_2024_4069_MOESM1_ESM.pdf]

Strain	TA98	TA100	TA1535	TA1537	WP2 *uvrA*
			-S9, Plate Incorporation		
Mean ± SD	5.0 ± 0.5	11.4 ± 0.7	4.3 ± 0.6	3.9 ± 0.5	4.1 ± 0.8
Min - Max	3.5 - 6.3	9.7 - 13.4	2.8 - 6.8	2.2 - 5.6	1.4 - 6.0
95% CL	4.1 - 6.0	10.0 - 12.8	3.2 - 5.4	2.8 - 4.9	2.5 - 5.6
N	55	54	55	56	55
			-S9, Preincubation		
Mean ± SD	5.1 ± 0.6	11.4 ± 0.8	4.3 ± 0.5	3.8 ± 0.4	4.4 ± 1.0
Min - Max	3.0 - 7.2	9.5 - 13.9	2.8 - 6.2	2.0 - 5.5	2.2 - 6.3
95% CL	3.9 - 6.2	9.8 - 13.0	3.2 - 5.4	2.9 - 4.7	2.5 - 6.4
N	46	46	46	46	47
			+S9, Plate Incorporation		
Mean ± SD	5.9 ± 0.5	11.6 ± 0.8	4.3 ± 0.6	4.3 ± 0.5	5.3 ± 0.9
Min - Max	4.1 - 7.5	9.9 - 13.7	2.8 - 6.3	2.0 - 5.7	3.2 - 7.9
95% CL	4.8 - 7.0	10.0 - 13.2	3.1 - 5.5	3.3 - 5.2	3.5 - 7.2
N	54	54	54	54	54
			+S9, Preincubation		
Mean ± SD	6.0 ± 0.6	11.6 ± 0.8	4.2 ± 0.4	4.2 ± 0.5	5.5 ± 1.0
Min - Max	4.5 - 8.0	9.5 - 13.7	3.0 - 5.7	2.8 - 5.9	2.8 - 7.6
95% CL	4.8 - 7.2	9.9 - 13.2	3.3 - 5.0	3.2 - 5.2	3.6 - 7.5
N	46	46	46	46	46
			Spontaneous Reversion		
Mean ± SD	5.1 ± 0.4	11.6 ± 0.8	4.3 ± 0.5	3.8 ± 0.5	4.3 ± 0.8
Min - Max	3.9 - 6.9	9.6 - 13.8	2.6 - 6.2	2.0 - 5.6	2.2 - 6.2
95% CL	4.2 - 6.0	10.0 - 13.1	3.4 - 5.3	2.9 - 4.7	2.7 - 5.9
N	76	74	75	76	76

A1: The Relative (Rel.) %RET for K21 at 500, 1000 and 2000 mg/kg at 36-45 hours for the male and female rats (**Appendix I).** At 60-72 hours, the Rel. %RET for K21 at 2000 mg/kg was 155.0% and 70.4% for males and females, respectively.
