# Supplementary material for: Newly discovered clouting interplay between matrix metalloproteinases structures and novel quaternary Ammonium K21: computational and in-vivo testing
Source: BMC Oral Health. 2024 Mar 25;24:382. doi: 10.1186/s12903-024-04069-0 (PMC10964527; doi:10.1186/s12903-024-04069-0)
Supplement: Supplementary file 2 — Supplementary Material 2. [file 12903_2024_4069_MOESM2_ESM.pdf]

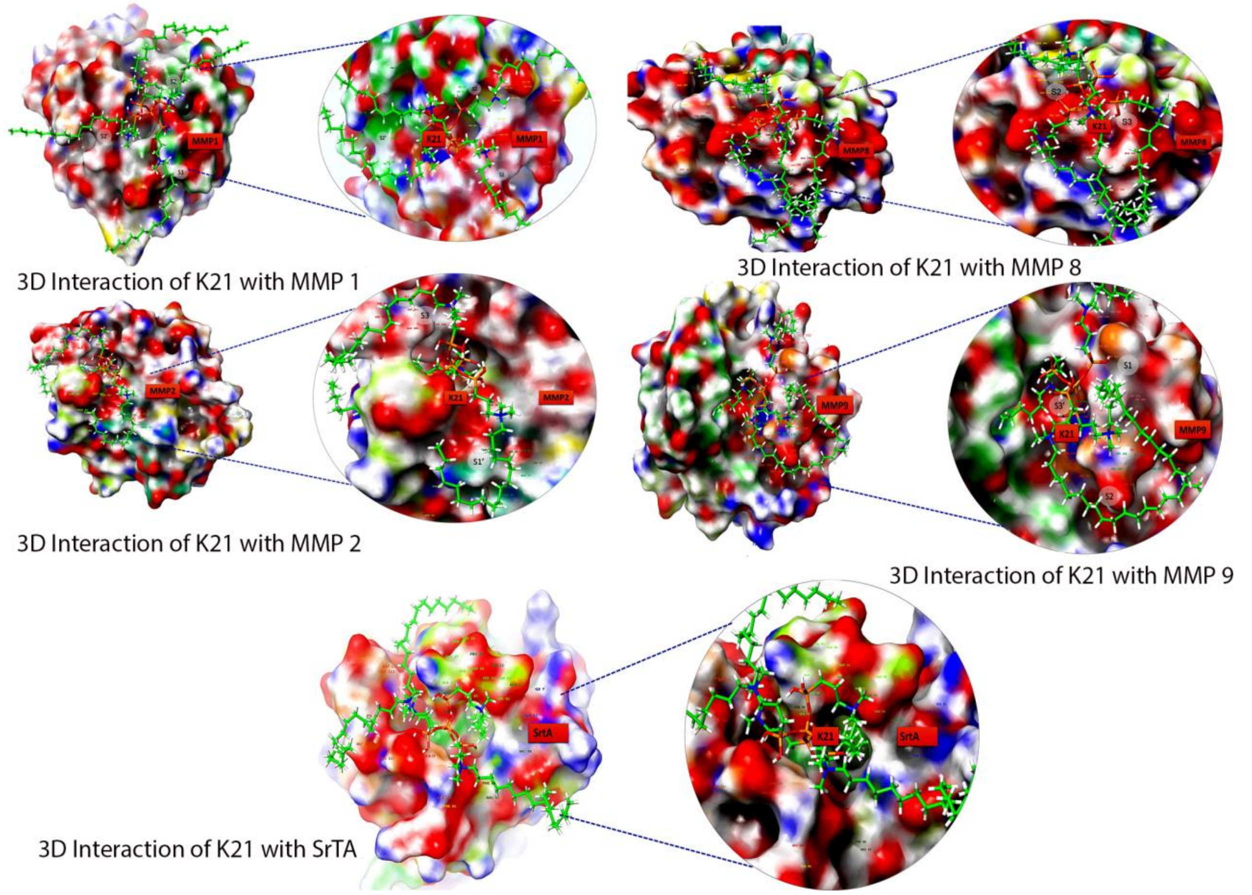

S1: 3D interaction of k21 attachment on MMP1, MMP2, MMP8, MMP9 and SrtA. As per the three-dimensional (3D) image analysis, K21 binds onto most active sites of unprimed and primed pockets of MMPs and exerts a *"clouting motion effect"* covering a larger attached portion on MMPs preventing attachment of other substrates.
